# Modern Management of Common Bile Duct Stones: Breakthroughs, Challenges, and Future Perspectives

**DOI:** 10.7759/cureus.75246

**Published:** 2024-12-06

**Authors:** Yanguang Sha, Zhilin Wang, Rongmei Tang, Ke Wang, Chen Xu, Guangbin Chen

**Affiliations:** 1 Graduate School, Wannan Medical College, Wuhu, CHN; 2 Department of Hepatobiliary Surgery, The Second People's Hospital of Wuhu, Wuhu Hospital Affiliated to East China Normal University, Wuhu, CHN

**Keywords:** biliary stone disease, choledocholithiasis, endoscopic retrograde cholangiopancreatography, laparoscopic common bile duct exploration, minimally invasive surgery, personalized medicine

## Abstract

Common bile duct (CBD) stone management has evolved significantly with technological advances and an improved understanding of pathophysiology. This comprehensive review examines current evidence and emerging trends in CBD stone management, emphasizing modern diagnostic approaches and treatment paradigms. Recent developments in imaging techniques, including AI-assisted analysis, have enhanced diagnostic accuracy. Treatment strategies now emphasize minimally invasive approaches, with endoscopic techniques showing success rates exceeding 90% in experienced centers. Special considerations for specific populations, including elderly patients and those with altered anatomy, have led to refined management algorithms. Future directions include novel stone fragmentation technologies, biodegradable materials, and personalized medicine approaches. The integration of these advances, combined with a multidisciplinary approach, has improved patient outcomes while presenting new opportunities for enhanced care delivery. Continued technological innovation and refined techniques suggest a promising future for CBD stone management, although challenges remain in optimizing treatment selection and preventing recurrence.

## Introduction and background

Common bile duct (CBD) stones represent a significant healthcare challenge in our aging global population, with substantial clinical and economic implications. The prevalence of gallstone disease affects up to 30% of individuals over 70 years, with CBD stones occurring in 10-20% of these cases [1,2]. Recent epidemiological data [3] indicates an incidence of acute cholangitis at 7.0 per 10,000 people, carrying a concerning mortality rate of up to 10%. Furthermore, the Global Burden of Disease Study 2019 [4] projects a significant increase in disease burden over the next decade, primarily driven by demographic shifts toward an aging population. These trends underscore the pressing need for innovative and effective management strategies to address this growing healthcare challenge.

The evolution of CBD stone management represents one of the most significant paradigm shifts in hepatobiliary surgery. Traditional open surgical procedures, once associated with substantial morbidity and mortality, have largely given way to minimally invasive approaches. The introduction and refinement of endoscopic retrograde cholangiopancreatography (ERCP) and laparoscopic techniques have transformed the therapeutic landscape, offering patients safer and more effective treatment options [5]. This transformation reflects not only technological advancement but also our enhanced understanding of CBD stone pathophysiology and an unwavering commitment to improving patient outcomes.

The contemporary approach to CBD stone management is driven by several compelling factors. First, demographic trends showing an aging population with increasing comorbidities necessitate less invasive treatment modalities that minimize procedural complications [6]. Second, rapid technological advancement has enabled more precise diagnostic techniques and therapeutic interventions, allowing for tailored treatment strategies. Third, the growing emphasis on patient-centered care has highlighted the importance of quality-of-life considerations and individualized treatment planning [7]. These factors collectively shape modern management approaches, which prioritize minimally invasive techniques, enhanced recovery protocols, and personalized treatment strategies [6].

The field of CBD stone management currently stands at a critical intersection of clinical necessity, technological innovation, and evolving patient expectations. While significant progress has been made in diagnostic capabilities and therapeutic options, important challenges persist, particularly in risk stratification, management of special patient populations, and optimization of economic outcomes [8-10]. This comprehensive review aims to synthesize current evidence, highlight recent breakthroughs, and examine emerging challenges and future perspectives in CBD stone management. By analyzing these aspects, we seek to provide clinicians with an updated framework for evidence-based decision-making and optimal patient care delivery.

## Review

Pathophysiology and risk factors

Stone Formation Mechanisms

Choledocholithiasis represents a complex pathophysiological process that manifests through two distinct mechanisms of stone formation. Primary CBD stones develop de novo within the biliary system due to bile stasis [11]. This condition typically occurs in settings of increased bile duct diameter, presence of biliary diverticula, or conditions predisposing to bacterial contamination of bile. In contrast, secondary CBD stones originate from gallbladder stones that migrate into the CBD, usually precipitated by gallbladder contractions and anatomical factors [11].

Clinical Complications

The pathophysiological cascade following stone formation involves several key mechanisms that can lead to significant clinical complications. Biliary obstruction results in increased intraductal pressure, creating biochemical and mechanical alterations of bile flow [12]. This obstruction commonly manifests as obstructive jaundice, bacterial colonization leading to bactibilia, ascending cholangitis, or gallstone pancreatitis [13,14]. These complications arise from the complex interplay between mechanical obstruction, inflammatory responses, and bacterial contamination of the biliary system.

Risk Factors

Multiple risk factors contribute to CBD stone formation, which can be categorized as modifiable and non-modifiable. Non-modifiable risk factors include advanced age, female gender, genetic predisposition (particularly ABCB4 gene mutations) [15], and anatomical variations in the biliary tract [16]. Modifiable risk factors encompass obesity, metabolic syndrome components, rapid weight loss, dietary factors (high-fat, low-fiber diet) [17], and physical inactivity. Special clinical conditions such as cirrhosis, primary sclerosing cholangitis, history of biliary surgery, total parenteral nutrition, and pregnancy also significantly increase the risk of stone formation [18].

Recurrence Risk Factors

Recent meta-analyses have identified specific risk factors for stone recurrence, which have important implications for long-term management [19]. These include CBD diameter ≥15 mm, sharp CBD angulation (<145 degrees), multiple ERCP interventions, postoperative pneumobilia, previous CBD surgery, and biliary stent placement history [20,21]. Understanding these pathophysiological mechanisms and risk factors has crucial implications for clinical management, influencing both diagnostic and therapeutic approaches. This knowledge guides risk stratification for diagnostic modality selection, determines intervention urgency, and influences the choice between different treatment techniques. Furthermore, it enables the implementation of targeted preventive measures for high-risk patients, guides post-procedure surveillance protocols, and informs patient education and lifestyle modification recommendations.

Modern diagnostic approaches

Clinical Assessment and Laboratory Studies

The accurate diagnosis of CBD stones requires a systematic, multimodal approach that balances diagnostic accuracy with cost-effectiveness and patient safety [22,23]. Modern diagnostic strategies employ a stepwise methodology, beginning with clinical assessment and laboratory studies before progressing to various imaging modalities. This integrated approach has demonstrated superior diagnostic accuracy while minimizing unnecessary invasive procedures.

Laboratory tests serve as crucial initial diagnostic tools in the evaluation of suspected CBD stones. Key biochemical markers include gamma-glutamyl transpeptidase, alkaline phosphatase, and total bilirubin, which serve as independent predictors of CBD stones [24]. These markers not only aid in initial diagnosis but also provide valuable information about the degree of biliary obstruction and cholestasis. Serial measurements of these parameters help monitor disease progression and treatment response, with the combination of markers providing diagnostic sensitivity and specificity [25].

Imaging Modalities

Imaging modalities play a central role in confirming the diagnosis and planning therapeutic interventions. Ultrasound (US) typically serves as the first-line imaging technique due to its widespread availability, cost-effectiveness, and non-invasive nature [26]. At the same time, US can identify CBD dilation and stones, characterized by echogenic rounded structures with posterior acoustic shadowing, with limitations in obese patients or those with interfering bowel gas [27]. Magnetic resonance cholangiopancreatography (MRCP) has emerged as the gold standard non-invasive imaging modality, offering exceptional accuracy with sensitivity rates of 85-98% and specificity of 75-96% [28-30]. MRCP provides detailed visualization of the biliary tree without the need for invasive procedures or contrast administration, making it particularly valuable for pre-procedure planning and anatomical mapping.

Endoscopic US (EUS) represents an advanced diagnostic tool that bridges the gap between imaging and intervention. EUS demonstrates remarkable accuracy, with detection rates of 96% for stones smaller than 5 mm and 98% for larger stones [31]. This modality offers the unique advantage of real-time imaging and the capability for guided intervention, making it particularly valuable in cases where traditional imaging results are equivocal or when anatomical variations complicate diagnosis. The integration of AI in medical imaging analysis has further enhanced diagnostic capabilities, with machine learning algorithms improving detection accuracy and enabling more precise stone measurements [32].

Diagnostic Algorithm and Selection Criteria

The selection of diagnostic modalities should be guided by a careful consideration of pre-test probability, cost-effectiveness, local expertise, and patient-specific factors [33]. For instance, an initial US followed by MRCP proves to be the most cost-effective in intermediate-risk patients, while direct EUS may be more appropriate in high-risk cases [22]. This tailored approach to diagnosis, incorporating both traditional and emerging technologies, has led to improved accuracy in CBD stone detection, reduced unnecessary invasive procedures, and better patient outcomes. The continued integration of AI and advanced imaging techniques promises even greater precision in diagnosis, ultimately supporting more effective treatment planning and improved clinical outcomes [34].

Current treatment paradigm

Pre-Intervention Assessment

The management of CBD stones has evolved significantly, emphasizing minimally invasive techniques and personalized treatment strategies. Modern treatment approaches begin with comprehensive patient evaluation, considering factors such as comorbidities, stone characteristics, and anatomical considerations [35]. This systematic assessment enables clinicians to select the most appropriate intervention while minimizing potential complications and optimizing outcomes.

Endoscopic Approaches

ERCP remains the cornerstone of CBD stone management, demonstrating impressive success rates of 85-98% for stone clearance [28-30]. Recent innovations in ERCP techniques have further enhanced its therapeutic potential. Endoscopic papillary balloon dilation has emerged as a valuable alternative to traditional sphincterotomy, particularly beneficial for large stones, as it reduces the need for mechanical lithotripsy and potentially lowers overall complications [36]. Additionally, cholangioscopy-assisted lithotripsy has proven effective in cases where conventional ERCP approaches fail, offering direct stone visualization and targeted fragmentation capabilities [36].

Surgical Management

Surgical management has undergone significant refinement with the advancement of minimally invasive techniques. Laparoscopic CBD exploration has gained prominence due to its excellent outcomes in terms of morbidity and mortality, while potentially reducing the need for multiple procedures [37]. This approach has demonstrated particular value in cases where ERCP is unsuccessful or contraindicated [38]. The emergence of robotic-assisted surgery has further expanded surgical capabilities, offering enhanced visualization and precision, which is especially beneficial in anatomically complex cases. These technological advancements have contributed to improved surgical outcomes and shorter recovery times [38].

Alternative Interventional Approaches

Alternative interventional approaches have been developed for cases where traditional methods may not be suitable. Percutaneous transhepatic cholangiography drainage has emerged as a viable option for high-risk patients or when other approaches fail, demonstrating technical success rates of 90-95% [39]. Novel interventional techniques, including EUS-guided approaches, have expanded treatment options, particularly valuable for patients with surgically altered anatomy [40]. These alternative approaches ensure that even complex cases can be managed effectively, although they typically require careful patient selection and expertise [41].

Multidisciplinary Team Approach

The success of modern CBD stone management relies heavily on a multidisciplinary team approach, particularly crucial for complex cases and recurrent stones. This collaborative framework typically involves hepatobiliary surgeons, interventional gastroenterologists, and radiologists working in concert to develop comprehensive treatment strategies [42]. The team approach enables more nuanced decision-making, considering factors such as patient comorbidities, stone characteristics, and anatomical considerations, ultimately leading to improved outcomes and reduced complications [43]. This integrated approach, combined with ongoing technological advancements and refined techniques, has transformed CBD stone management into a more precise and patient-centered discipline.

Special considerations

Elderly Patients

The management of CBD stones requires careful modification of standard approaches in certain patient populations and clinical scenarios. Elderly patients, particularly those over 80 years of age, present unique challenges due to increased comorbidities and altered physiological reserves [44]. In this population, endoscopic management with ERCP demonstrates favorable outcomes, with technical success rates of 90-95%, although careful patient selection and modified techniques are essential to minimize complications [39]. Pre-procedure optimization, including careful assessment of coagulation status and cardiopulmonary function, significantly influences outcomes [45]. Recent studies have shown that abbreviated anticoagulation bridges and minimal sedation protocols can reduce procedure-related complications while maintaining therapeutic efficacy [45].

Pregnancy Management

Pregnancy presents another challenging scenario requiring specialized management approaches. CBD stones during pregnancy carry significant risks for both mother and fetus, necessitating careful consideration of diagnostic and therapeutic options. While MRCP without gadolinium remains the preferred diagnostic tool, therapeutic interventions must be tailored to gestational age and severity of presentation [46]. ERCP, when necessary, should be performed with minimal fluoroscopy time and appropriate fetal shielding, preferably during the second trimester [47]. Studies have demonstrated successful outcomes with modified ERCP techniques, reporting maternal and fetal complication rates comparable to non-pregnant patients when performed by experienced endoscopists [48].

Altered Surgical Anatomy

Patients with altered surgical anatomy, particularly those who have undergone Roux-en-Y gastric bypass or hepaticojejunostomy, require specialized approaches to CBD stone management. Traditional ERCP may be technically challenging or impossible in these cases, necessitating alternative strategies. Device-assisted enteroscopic ERCP has become a viable option, with a high technical success rate in experienced centers [49]. When enteroscopy-assisted approaches fail, percutaneous or surgical interventions may be necessary. Recent advances in EUS-guided techniques have expanded treatment options for these challenging cases, although such approaches should be reserved for centers with appropriate expertise [9].

Complex Stones

Complex stones, defined by size, location (intrahepatic), or composition (hard consistency), require modified therapeutic strategies. These cases often benefit from a combination of approaches, including cholangioscopy-assisted lithotripsy, large balloon dilation, or novel fragmentation techniques [50,51]. The management strategy should consider not only immediate stone clearance but also the prevention of recurrence, particularly important in patients with intrahepatic stones or primary stone formation. Long-term outcomes in these cases are optimized through careful follow-up protocols and preventive measures, including ursodeoxycholic acid therapy when indicated [52].

Acute Cholangitis Management

The management of acute cholangitis accompanying CBD stones requires particular attention to the timing and sequence of interventions. Early recognition and risk stratification using validated scoring systems (such as Tokyo Guidelines) guide the urgency and nature of intervention [53]. While emergency decompression is essential in severe cases, the timing of definitive stone clearance should be individualized based on patient stability and local expertise. Recent evidence supports early ERCP (within 24-48 hours) in moderate to severe cases while suggesting that stable patients with mild cholangitis may safely undergo delayed intervention after appropriate antibiotic therapy [54].

**Figure 1:**
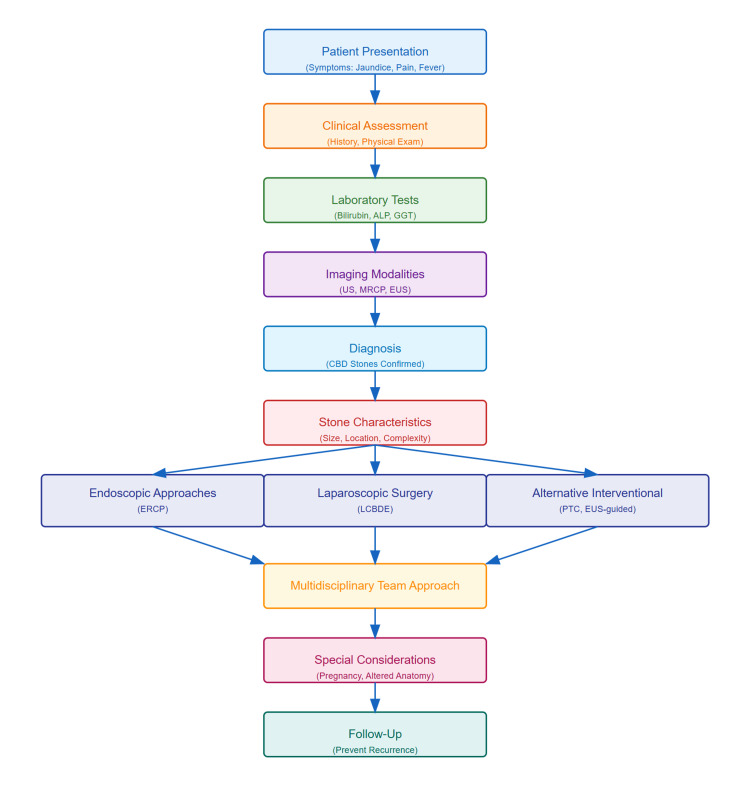
Management algorithm for CBD stones: a comprehensive approach Initial evaluation begins with a clinical assessment of primary symptoms (jaundice, pain, and fever), followed by laboratory tests (bilirubin, ALP, and GGT) and imaging studies (US as first-line, MRCP as gold standard, and EUS for special cases). Treatment selection is primarily based on stone characteristics (size, location, and complexity): endoscopic approaches (ERCP) and laparoscopic surgery serve as the primary intervention and alternative interventional approaches are reserved for special anatomical considerations or complex cases. Special attention should be given to elderly patients (preferring minimally invasive approaches), pregnancy (requiring careful timing), and anatomical variations (potentially needing alternative interventions). A multidisciplinary team approach is essential for complex cases, recurrent stones, and treatment failures. Long-term follow-up focuses on monitoring and preventing recurrence. Treatment decisions should be individualized based on patient characteristics, institutional expertise, and available resources, emphasizing the importance of a comprehensive, patient-centered approach to optimize outcomes. ALP: alkaline phosphatase; CBD: common bile duct; ERCP: endoscopic retrograde cholangiopancreatography; EUS: endoscopic ultrasound; GGT: gamma-glutamyl transferase; MRCP: magnetic resonance cholangiopancreatography; US: ultrasound Image credit: Guangbin Chen

The algorithm (Figure 1) illustrates the standardized diagnostic and therapeutic approach for CBD stones. This enhanced understanding of special clinical scenarios has led to more nuanced and effective management strategies. Success in these challenging cases relies heavily on appropriate patient selection, modification of standard techniques, and careful attention to timing and sequence of interventions.

Future directions

AI Applications

The management of CBD stones continues to evolve rapidly, driven by technological innovations and improved understanding of disease pathophysiology. AI and machine learning applications represent one of the most promising developments in this field [55]. These technologies are being integrated into various aspects of CBD stone management, from automated stone detection in imaging studies to the prediction of treatment outcomes. Early studies demonstrate that AI-assisted systems can achieve detection accuracy rates exceeding 90% [56]. Furthermore, machine learning algorithms are being developed to predict stone recurrence risk and optimize personalized treatment strategies, potentially revolutionizing our approach to long-term management [57].

Technological Innovations

Technological advances in minimally invasive procedures are reshaping therapeutic approaches to CBD stones. Novel stone fragmentation technologies, including laser systems with enhanced precision and reduced tissue damage, are showing promising results in initial trials [58]. These systems, combined with improved visualization technologies, enable more effective stone clearance while minimizing complications. The development of biodegradable stents and smart materials represents another exciting frontier, potentially offering temporary biliary drainage without the need for subsequent removal procedures [59]. Early clinical trials of these materials have demonstrated encouraging results, with biodegradation profiles matching clinical requirements and minimal inflammatory responses.

Robotic Developments

Robotic assistance in biliary procedures is advancing rapidly, with newer platforms offering enhanced precision and improved ergonomics. These systems are being adapted for both endoscopic and surgical approaches, potentially expanding the scope of minimally invasive interventions [60]. Initial studies suggest that robotic assistance may particularly benefit complex cases, such as those involving intrahepatic stones or altered anatomy [61]. While current cost considerations limit widespread adoption, ongoing technological refinements and increasing competition are expected to improve accessibility. The integration of haptic feedback and enhanced imaging capabilities in these systems promises to further improve procedural outcomes.

Prevention Strategies

Research into the prevention of stone formation and recurrence continues to yield important insights. Molecular studies are identifying new therapeutic targets, particularly in the pathways involved in cholesterol metabolism and bile composition. Novel medical therapies, including modified bile acids and targeted anti-inflammatory agents, are under investigation for their potential role in preventing stone formation and reducing recurrence rates [62]. Additionally, research into the gut microbiome’s role in stone formation may lead to innovative preventive strategies, including targeted probiotic therapies or microbiome modification approaches [63].

Personalized Medicine Approaches

The future of CBD stone management also emphasizes personalized medicine approaches. Advanced imaging techniques, including molecular imaging and novel contrast agents, are being developed to better characterize stone composition and predict treatment response [64]. Genetic studies are identifying markers associated with stone formation risk and treatment outcomes, potentially enabling more targeted preventive strategies [65]. The integration of these personalized approaches with existing treatment modalities promises to improve patient outcomes while optimizing resource utilization.

Quality Metrics and Outcome Measures

Quality metrics and outcome measures continue to evolve, with increasing emphasis on patient-reported outcomes and long-term results [66]. International collaborations are establishing standardized reporting systems and quality benchmarks, facilitating more meaningful comparisons across institutions and treatment approaches [67]. These efforts, combined with the development of comprehensive databases and registries, will provide valuable insights into optimal management strategies and help identify areas for improvement.

## Conclusions

The management of CBD stones has undergone a remarkable transformation, driven by technological advances and an improved understanding of disease pathophysiology. Modern management emphasizes a systematic approach to diagnosis and treatment, incorporating advanced imaging modalities, enhanced endoscopic techniques, and novel surgical approaches. ERCP remains the cornerstone of therapy, while the development of cholangioscopy-assisted lithotripsy and minimally invasive surgical techniques has expanded options for challenging cases. The integration of AI in imaging analysis and the evolution of treatment modalities have particularly benefited specific patient populations, including elderly patients and those with complex anatomical variations.

These developments carry significant implications for clinical practice, emphasizing the importance of personalized treatment strategies and multidisciplinary collaboration. Success in managing CBD stones requires careful patient selection, appropriate application of available technologies, and consideration of cost-effectiveness. While challenges remain in managing complex cases and preventing recurrence, ongoing technological advances and refined techniques suggest a promising future. The key to optimal outcomes lies in balancing innovative approaches with practical considerations, maintaining a focus on evidence-based practice and patient-centered care. As the field continues to evolve, healthcare providers must stay current with emerging technologies while preserving expertise in fundamental techniques, ensuring the best possible outcomes for patients affected by this condition.

## References

[1] Williams E, Beckingham I, El Sayed G, Gurusamy K, Sturgess R, Webster G, Young T (2017). Updated guideline on the management of common bile duct stones (CBDS). Gut.

[2] Kazi FN, Ghosh S, Sharma JV, Saravanan S, Patil S (2022). Trends in gallbladder disease in young adults: a growing concern. Cureus.

[3] Tan M, Schaffalitzky de Muckadell OB, Laursen SB (2019). Unchanged mortality in patients with acute cholangitis despite an increase in malignant etiologies - a 25-year epidemiological study. Scand J Gastroenterol.

[4] (2020). Global burden of 369 diseases and injuries in 204 countries and territories, 1990-2019: a systematic analysis for the Global Burden of Disease Study 2019. Lancet.

[5] Buxbaum JL, Abbas Fehmi SM, Sultan S (2019). ASGE guideline on the role of endoscopy in the evaluation and management of choledocholithiasis. Gastrointest Endosc.

[6] Chhoda A, Mukewar SS, Mahadev S (2021). Managing gallstone disease in the elderly. Clin Geriatr Med.

[7] Gao J, Ding XM, Ke S (2013). Anisodamine accelerates spontaneous passage of single symptomatic bile duct stones ≤ 10 mm. World J Gastroenterol.

[8] Manes G, Paspatis G, Aabakken L (2019). Endoscopic management of common bile duct stones: European Society of Gastrointestinal Endoscopy (ESGE) guideline. Endoscopy.

[9] Tringali A, Costa D, Fugazza A, Colombo M, Khalaf K, Repici A, Anderloni A (2021). Endoscopic management of difficult common bile duct stones: where are we now? A comprehensive review. World J Gastroenterol.

[10] Mattila A, Mrena J, Kellokumpu I (2017). Cost-analysis and effectiveness of one-stage laparoscopic versus two-stage endolaparoscopic management of cholecystocholedocholithiasis: a retrospective cohort study. BMC Surg.

[11] Cianci P, Restini E (2021). Management of cholelithiasis with choledocholithiasis: endoscopic and surgical approaches. World J Gastroenterol.

[12] Cozma MA, Găman MA, Srichawla BS (2024). Acute cholangitis: a state-of-the-art review. Ann Med Surg (Lond).

[13] Moon DK, Kang JS, Byun Y, Choi YJ, Lee HW, Jang JY, Lim CS (2023). Incidence of bactibilia and related factors in patients who undergo cholecystectomy. Ann Surg Treat Res.

[14] Lavillegrand JR, Mercier-Des-Rochettes E, Baron E (2021). Acute cholangitis in intensive care units: clinical, biological, microbiological spectrum and risk factors for mortality: a multicenter study. Crit Care.

[15] Mansour S, Kluger Y, Khuri S (2022). Primary recurrent common bile duct stones: timing of surgical intervention. J Clin Med Res.

[16] Wang SF, Wu CH, Sung KF (2023). The impact of metabolic factors and lipid-lowering drugs on common bile duct stone recurrence after endoscopic sphincterotomy with following cholecystectomy. J Pers Med.

[17] Hoyas I, Leon-Sanz M (2019). Nutritional challenges in metabolic syndrome. J Clin Med.

[18] Saito H, Tada S (2022). Risk prediction of common bile duct stone recurrence based on new common bile duct morphological subtypes. World J Gastrointest Surg.

[19] Wen N, Wang Y, Cai Y (2023). Risk factors for recurrent common bile duct stones: a systematic review and meta-analysis. Expert Rev Gastroenterol Hepatol.

[20] Choe JW, Kim SY, Lee DW (2021). Incidence and risk factors for postoperative common bile duct stones in patients undergoing endoscopic extraction and subsequent cholecystectomy. Gastrointest Endosc.

[21] Kim JH, Kim YS, Kim DK (2011). Short-term clinical outcomes based on risk factors of recurrence after removing common bile duct stones with endoscopic papillary large balloon dilatation. Clin Endosc.

[22] Morris S, Gurusamy KS, Sheringham J, Davidson BR (2015). Cost-effectiveness analysis of endoscopic ultrasound versus magnetic resonance cholangiopancreatography in patients with suspected common bile duct stones. PLoS ONE.

[23] Ebrahim M, Sorensen LT, Jorgensen LN, Kalaitzakis E (2018). Current clinical algorithms for predicting common bile duct stones have only moderate accuracy. Dig Endosc.

[24] Copelan A, Kapoor BS (2015). Choledocholithiasis: diagnosis and management. Tech Vasc Interv Radiol.

[25] Krupa Ł, Staroń R, Dulko D (2021). Importance of bile composition for diagnosis of biliary obstructions. Molecules.

[26] Badea R, Crisan M (2024). The utility of ultrasound for the diagnosis and treatment of primary and metastatic melanoma - a remaining oncological challenge. Med Ultrason.

[27] Heinitz S, Müller J, Jenderka KV (2023). The application of high-performance ultrasound probes increases anatomic depiction in obese patients. Sci Rep.

[28] Makmun D, Fauzi A, Shatri H (2017). Sensitivity and specificity of magnetic resonance cholangiopancreatography versus endoscopic ultrasonography against endoscopic retrograde cholangiopancreatography in diagnosing choledocholithiasis: the Indonesian experience. Clin Endosc.

[29] Isram J, Haider E, Khan RS (2023). Diagnostic accuracy of magnetic resonance cholangiopancreatography in comparison with endoscopic retrograde cholangiopancreatography for detection of the etiology of obstructive jaundice. Cureus.

[30] Suzuki M, Sekino Y, Hosono K (2022). Endoscopic ultrasound versus magnetic resonance cholangiopancreatography for the diagnosis of computed tomography-negative common bile duct stone: prospective randomized controlled trial. Dig Endosc.

[31] Meeralam Y, Al-Shammari K, Yaghoobi M (2017). Diagnostic accuracy of EUS compared with MRCP in detecting choledocholithiasis: a meta-analysis of diagnostic test accuracy in head-to-head studies. Gastrointest Endosc.

[32] Khalifa M, Albadawy M (2024). AI in diagnostic imaging: revolutionising accuracy and efficiency. Comput Methods Programs Biomed.

[33] De Angelis CG, Dall'Amico E, Staiano MT (2023). The endoscopic retrograde cholangiopancreatography and endoscopic ultrasound connection: unity is strength, or the endoscopic ultrasonography retrograde cholangiopancreatography concept. Diagnostics (Basel).

[34] Rong J, Liu Y (2024). Advances in medical imaging techniques. BMC Methods.

[35] Sharma A, Dahiya P, Khullar R, Soni V, Baijal M, Chowbey PK (2012). Management of common bile duct stones in the laparoscopic era. Indian J Surg.

[36] Franzini T, Moura RN, Bonifácio P (2018). Complex biliary stones management: cholangioscopy versus papillary large balloon dilation - a randomized controlled trial. Endosc Int Open.

[37] Dagnino G, Kundrat D (2024). Robot-assistive minimally invasive surgery: trends and future directions. Int J Intell Robot Appl.

[38] Alkhamesi NA, Davies WT, Pinto RF, Schlachta CM (2013). Robot-assisted common bile duct exploration as an option for complex choledocholithiasis. Surg Endosc.

[39] Chandrashekhara SH, Gamanagatti S, Singh A, Bhatnagar S (2016). Current status of percutaneous transhepatic biliary drainage in palliation of malignant obstructive jaundice: a review. Indian J Palliat Care.

[40] Sharaiha RZ, Khan MA, Kamal F (2017). Efficacy and safety of EUS-guided biliary drainage in comparison with percutaneous biliary drainage when ERCP fails: a systematic review and meta-analysis. Gastrointest Endosc.

[41] Sharma M, Hollerbach S, Fusaroli P (2021). General principles of image optimization in EUS. Endosc Ultrasound.

[42] Oh JH, Sinn DH (2024). Multidisciplinary approach for hepatocellular carcinoma patients: current evidence and future perspectives. J Liver Cancer.

[43] Liang W, Qin G, Yu L, Wang Y (2023). Reducing complications of femoral neck fracture management: a retrospective study on the application of multidisciplinary team. BMC Musculoskelet Disord.

[44] Tarikci Kilic E, Kahraman R, Ozdil K (2019). Evaluation of safety and outcomes of endoscopic retrograde cholangiopancreatography in 1337 patients at a single center. Medeni Med J.

[45] D'Andrilli A, Massullo D, Rendina EA (2018). Enhanced recovery pathways in thoracic surgery from Italian VATS Group: preoperative optimisation. J Thorac Dis.

[46] Neuhaus H (2020). Choledocholithiasis in pregnancy: when and how to perform ERCP?. Endosc Int Open.

[47] Dumonceau JM, Garcia-Fernandez FJ, Verdun FR (2012). Radiation protection in digestive endoscopy: European Society of Digestive Endoscopy (ESGE) guideline. Endoscopy.

[48] Azab M, Bharadwaj S, Jayaraj M (2019). Safety of endoscopic retrograde cholangiopancreatography (ERCP) in pregnancy: a systematic review and meta-analysis. Saudi J Gastroenterol.

[49] Nehme F, Goyal H, Perisetti A, Tharian B, Sharma N, Tham TC, Chhabra R (2021). The evolution of device-assisted enteroscopy: from sonde enteroscopy to motorized spiral enteroscopy. Front Med (Lausanne).

[50] Karagyozov P, El-Atrebi K, Boeva I, Tishkov I (2023). Cholangioscopy-guided lithotripsy in the treatment of difficult bile ducts stones - Bulgarian and Egyptian experience. Folia Med (Plovdiv).

[51] Murabayashi T, Kanno Y, Koshita S (2020). Long-term outcomes of endoscopic papillary large-balloon dilation for common bile duct stones. Intern Med.

[52] Hormati A, Ghadir MR, Sarkeshikian SS (2020). Adding ursodeoxycholic acid to the endoscopic treatment and common bile duct stenting for large and multiple biliary stones: will it improve the outcomes?. BMC Gastroenterol.

[53] Miura F, Okamoto K, Takada T (2018). Tokyo Guidelines 2018: initial management of acute biliary infection and flowchart for acute cholangitis. J Hepatobiliary Pancreat Sci.

[54] Park N, Lee SH, You MS (2021). Optimal timing of endoscopic retrograde cholangiopancreatography for acute cholangitis associated with distal malignant biliary obstruction. BMC Gastroenterol.

[55] Lee LI, Kanthasamy S, Ayyalaraju RS, Ganatra R (2019). The current state of artificial intelligence in medical imaging and nuclear medicine. BJR Open.

[56] Chen M, Wang Y, Wang Q (2024). Impact of human and artificial intelligence collaboration on workload reduction in medical image interpretation. NPJ Digit Med.

[57] Gulhane M, Kumar S, Choudhary S (2024). Integrative approach for efficient detection of kidney stones based on improved deep neural network architecture. SLAS Technol.

[58] Santa Cruz JA, Danilovic A, Vicentini FC (2024). Ureteral access sheath. Does it improve the results of flexible ureteroscopy? A narrative review. Int Braz J Urol.

[59] Zong J, He Q, Liu Y, Qiu M, Wu J, Hu B (2022). Advances in the development of biodegradable coronary stents: a translational perspective. Mater Today Bio.

[60] Lu J, Xu BB, Zheng HL (2024). Robotic versus laparoscopic distal gastrectomy for resectable gastric cancer: a randomized phase 2 trial. Nat Commun.

[61] Zhu G, Ifuku KA, Kirkwood KS (2023). Robotic-assisted approach for complex cholecystectomies. Mini Invasive Surg.

[62] Di Ciaula A, Portincasa P (2018). Recent advances in understanding and managing cholesterol gallstones. F1000Res.

[63] Yuan T, Xia Y, Li B (2023). Gut microbiota in patients with kidney stones: a systematic review and meta-analysis. BMC Microbiol.

[64] Mhlanga N, Mphuthi N, Van der Walt H, Nyembe S, Mokhena T, Sikhwivhilu L (2024). Nanostructures and nanoparticles as medical diagnostic imaging contrast agents: a review. Mater Today Chem.

[65] Alharbi SA, Alshenqiti AM, Asiri AH, Alqarni MA, Alqahtani SA (2023). The role of genetic testing in pediatric renal diseases: diagnostic, prognostic, and social implications. Cureus.

[66] Shahian DM, Meyer GS, Mort E (2012). Association of National Hospital Quality Measure adherence with long-term mortality and readmissions. BMJ Qual Saf.

[67] Chang HC, Tzou DT, Usawachintachit M (2016). Rationale and design of the registry for stones of the kidney and ureter (ReSKU): a prospective observational registry to study the natural history of urolithiasis patients. J Endourol.

